# Feasibility and preliminary effects of the mindful healthy family project among rural families

**DOI:** 10.1080/21642850.2024.2446368

**Published:** 2024-12-26

**Authors:** Tsui-Sui Annie Kao, Jiying Ling, Mohammed Alanazi, Nick Bara, Jessica Barnes Najor

**Affiliations:** a College of Nursing, Michigan State University, East Lansing, MI, USA; b University of Bisha, Bisha, Saudi Arabia; c College of Human Medicine, Michigan State University, East Lansing, MI, USA; d University Outreach and Engagement, Michigan State University, East Lansing, MI, USA

**Keywords:** Mindfulness, motivational interviewing, obesogenic behaviors, lifestyle modification

## Abstract

**Background/purpose:**

Rural adults and children are at higher risk for overweight and obesity. However, there are relatively few lifestyle modification programs available for these high-risk families, mainly because of the difficulty in reaching them. This mindfulness-based motivational interviewing (MM-based-MI) pilot aimed to improve parents’ healthy eating index (HEI), collective family efficacy, family satisfaction, perceived stress, and depressive symptoms as well as parent–child dyads’ eating patterns, physical activity (PA), and body mass index (BMI).

**Methods:**

This randomized controlled trial (RCT) was conducted in the Midwestern US to examine the feasibility (enrollment, attendance, and attrition), acceptability, and preliminary effects of an MM-based-MI intervention that contained nine sessions of health coaching (1-on-1), while the active-control included nine emailed health handouts sent over an 18-week period.

**Results:**

A total of 46 parents (29 intervention, 17 control; M_age _= 38.5 years, 85% female) participated. The enrollment rate, intervention attendance rate, and attrition rate were 12.6%, 79.8%, and 23.9% respectively. Although not statistically significant, compared to the control, the MM-based-MI intervention showed positive effects on improving parents’ HEI in terms of increasing total HEI score (Cohen’s *d *= 0.43), vegetable intake (*d *= 0.41), greens/beans (*d *= 0.59), protein food (*d *= 0.82), and self-efficacy in exercise (*d *= 0.21), as well as decreasing total calories (Kcal, *d *= −0.58), added sugar (*d *= −0.50), and depressive symptoms (*d *= −0.42), while controlling for marital status. Controlling for age, sex, and marital status, intervention children had greater improvement in increasing fiber (*d *= 0.75) and protein (*d *= 0.72) intake compared to the active-control group. Moreover, parents in both groups reported improvement (small to large effects) in ↑mindful eating, ↑collective family efficacy, ↑family satisfaction, and ↓perceived stress over time.

**Conclusions:**

Despite some limitations (small sample size, virtual at home measurement), our results support the feasibility, acceptability, and preliminary effects of this *Mindful Healthy Family* program on potentially mitigating some obesogenic behaviors among rural parent–child dyads.

**Trial registration:** ClinicalTrials.gov identifier: NCT05324969.

## Introduction

Obesogenic behaviors (poor dietary intake and inadequate physical activity [PA]) play an important role in an individual’s weight gain,(Filos et al., 2021) leading to increased risk of developing type 2 diabetes, cardiovascular diseases, cancers, depression, and contributing to overall poor quality of life (Weihrauch-Blüher et al., 2019). In the United States, the prevalence of adults with obesity rose from 33.8% in 2008–42.4% in 2018, with a notable increase in the rural population (Li & Whitacre, 2022). Compared with urban residents, rural adults and children are at increased risk (6.2 times higher prevalence) for overweight and obesity (Adams et al., 2012; McCormack & Meendering, 2016; Okobi et al., 2021). Factors that contribute to rural-urban disparities in obesity prevalence include differences in education level and the neighborhood-built environment (Wen et al., 2018). Yet, relatively few effective lifestyle modification programs have been designed specifically for rural adults or children. One of the biggest challenges is the difficulty in reaching these highly vulnerable people in an efficient manner. Virtual programs (telemedicine interventions) are a recommended approach to help reach adults who need weight management or obesity prevention services (Ufholz & Bhargava, 2021). However, to the best of our knowledge, no virtual interventions have been identified that focus on reducing obesogenic behaviors in rural populations.

It is plausible to combine motivational interviewing (MI) and mindfulness meditation (MM) for lifestyle modification because both are a) designed to promote behavioral self-management (theoretical congruency) (Benzo, 2013), b) ideal to serve as complementary tools to empower and sustain behavioral changes (Sohl et al., 2016), and c) easier for health care providers to accept and implement to mitigate health promotion-related stress (Bischof et al., 2021; Chmielewski et al., 2021). It is important to note that the MI plus Cognitive Behavioral Therapy is recognized as an optimal treatment for anxiety disorders (Randall & McNeil, 2017). However, providers generally require extensive training to use such a combination to reduce stress (Nakao et al., 2021).

The development of the MM-based-MI content (Table 1) was informed by our multiple preliminary works (Kao et al., 2021a; Kao et al., 2021c; Kao et al., 2023a; Kao et al., 2023b; Kao et al., 2023c). First, a nurse-led, three-session (in-person), stage-tailored MI program was well received by 23 primarily African American (52%) females who were overweight/obese (mean body mass index [BMI] = 39.2) and had prediabetes (Kao et al., 2021a). This small pilot resulted in significant decreases in participants’ waist circumferences and systolic*/*diastolic blood pressures as well as improvements in exercise self-efficacy, eating patterns, and PA, as well as readiness for behavioral changes (Kao et al., 2021a). In addition, the MI algorithm used in this study was implemented and found feasible with 24 parents of young children (2 sessions conducted 2 weeks apart) in terms of helping and supporting their children to adopt improved mindful eating behaviors (Smriti et al., 2022). The effectiveness of MI on children’s obesogenic behaviors was further confirmed in recent systematic reviews and meta-analyses in which the short-term effects of MI on children’s obesogenic behaviors and anthropometric outcomes (BMI and percent body fat) were significantly documented, with effect sizes ranging from small to medium (Kao et al., 2021c; Kao et al., 2023c). Moreover, the significant short  – and long-term effects of mindfulness-based interventions were established with pooled effect sizes (ranging from small to medium) on participating adults’ BMI and weight(Kao et al., 2023a) as well as eating behaviors (Kao et al., 2023b). Taken together, it is reasonable to use MI as an adjunct to an MM-based intervention for these lifestyle modifications because MI techniques can be used to elicit participants’ motivation toward making behavioral changes (addressing ambivalence), while MM practices can be utilized to alleviate stress (or anxiety) associated with intended changes (Lehto et al., 2022). Nonetheless, MM-based interventions have been found to reduce adults’ perceived stress (Nyklíček & Kuijpers, 2008), which is recognized as a significant contributor to obesogenic behaviors (Scott et al., 2012). Finally, recent advances in telehealth have provided a new channel for serving the hard-to-reach rural population (Jin et al., 2020). For example, in a systematic review with four articles, there was high satisfaction from patients regarding the use of telehealth for receiving health services (Harkey et al., 2020). For these reasons, it is logical to combine these two approaches (MM-based-MI) into a virtual program designed to mitigate rural parents’ obesogenic behaviors. Thus, the objectives of this randomized controlled trial (RCT) were to examine the feasibility (enrollment, attendance, and attrition), acceptability, and preliminary effects of a fully virtual MM-based-MI intervention, the *Mindful-Healthy Family,* on parents/caregivers’ (referred as parents thereafter) ↑Healthy Eating Index (HEI, the primary outcome), ↑collective family efficacy, ↑family satisfaction, ↓perceived stress, and ↓depressive symptoms as well as the parent–child dyads’ ↓BMI, ↑healthy eating patterns, and ↑PA.

**Table 1.** Content of Nine MM-based-MM sessions.

	MI Coaching	**MM Exercise**		
Session	↑Self-efficacy; ↑Commitment; ↑Goal Attaining	Mindful Eating and Movement	Mindful Interactions	Stress Reduction
#1	Stages of Change assessment (perceived importance, readiness, confidence); identify problem, strength, and motivator.	What is mindful eating? Introduce 7 types of hunger: Raisin exercise.	Enhance family communication via *Family Meeting*	Relaxing deep breathing instruction
#2	Identify personal, familial, cultural, or religious strengths. Discuss personal short – and long-term goals.	Explore eye, nose, and mouth hungers; 3 Yoga poses with breathing and reflection.	Discovering your own family strengths; exercise awareness.	Explore stress (good or bad); mini meditations with breathing
#3	Strategies to set/achieve short-term goals; assess education needs (*obtain permission to provide education*) and preferred way for obtaining educational material.	Hunger awareness (stomach, cellular). Two-plate approach; Explore Qigong and walking along with breathing.	Strategies to elicit support at home, school, community.	Explore ways to deal with good and bad stress. *Family temperatures.*
#4	Explore inner motivation; coaching to understand individual’s needs and goal (know why). Reappraise Stage of Change.	Explore mind hunger; food portion size and presentation; 3 more Yoga poses.	Effective parenting; My complimentary words and moments.	Body scan exercise with breathing.
#5	Goal setting and attainment; personal/family goals and plans.	Groceries vs. fast food (total cost and calories); Arm swinging exercise.	Establish family routine for healthy eating and activities.	Prioritizing my tasks (Exercise: *Today I will and Today I will not*).
#6	Develop achievable goals and action plan; determine personal/family action plan activities.	Mindful eating meditation learning tool; Exercise, movement awareness.	Positive thoughts; Self-affirmation/compassion.	Alternative strategies to handle frustration.
#7	Elicit strengths and resources; establish family routine and living with change; Becoming aware of others’ needs.	Belly breathing learning tool; making time for family mindfulness.	Family strengths check and utilization.	Compassion for self and others exercises.
#8	What strategies work and do not work? Develop mindfulness action plan.	Develop action plan for healthy eating and activities. Favorite exercise.	Family fun list and kid’s choice.	Bring mindfulness into everyday life.
#9	Finalize action plans for later; re-evaluate personal and family strengths and barriers.	Development of personal and familial mindfulness action plan.	Sustaining a healthy habit learning tool.	What healthy eating looks like learning tool.

### Theoretical frameworks

The Social Ecological Model (McCormick et al., 2021) and Family Resiliency Theory (Walsh, 2003) were used to guide the development of this *Mindful-Healthy Family* program. More specifically, the Social Ecological Model has been used to evaluate how parents may serve as a change agent for improving parent–child dyads’ lifestyle behaviors within the family system. This model recognizes multiple levels of influence on health behaviors and highlights people’s interaction with their physical and sociocultural environments (McCormick et al., 2021). Family Resiliency Theory was used to support how our pilot intervention may improve these parents’ collective family efficacy and family satisfaction and be protective against perceived stress, depression, and obesogenic behaviors (Walsh, 2003). Moreover, mindful parenting has been found to decrease clinical symptoms in children with attention deficit hyperactivity disorder and their parents’ anxiety level (Behbahani et al., 2018). Based on Family Resiliency Theory, this MM-based-MI intervention has the potential to improve mindful interactions between parents and children and decrease parents’ perceived stress, thereby diminishing the dyads’ obesogenic behaviors and resulting in lowering their BMI. Mindful eating, mindful movements, mindful interactions, and stress reduction strategies were delivered using MI techniques (Open questioning, Affirming, Reflecting, and Summarizing [OARS]) in a nonjudgmental manner (Kao et al., 2021a; Miller & Rollnick, 2013; Smriti et al., 2022). Although this pilot primarily targeted adult parents’ obesogenic behaviors, we have hypothesized that the positive parental effects may also be correlated with the effects noted in children since parents play a significant modeling role in the family system.

## Methods

### Study design and sample

A randomized clinical trial (RCT) was conducted from March 2022 to January 2024 in the Midwestern United States. Forty-six families living in rural communities were randomly assigned (stratified by overweight or obesity weight status) to the intervention (n = 29) and active-control (n = 17) groups, for a 2–1 ratio, respectively. The enrollment period spanned more than 18 months, from April 2022 to October 2023, and used a rolling ‘first-come-first-served’ enrollment design (Gupta et al., 2015). Michigan State University’s institutional review board approved the study. The trial was registered at ClinicalTrials.gov (NCT05324969).

Our inclusion criteria were parents who 1) resided in a rural community (verified by zip code and home address), 2) had a BMI between 25 and 45, 3) lived with a child aged 3‒11 years, 4) had a valid email address and access to zoom conference or a telephone, and 5) was willing to complete at-home weight and height assessments and online surveys. We excluded potential participants who had severe mental and physical health problems (a priori criterion; none was excluded as the result) or were pregnant at baseline. The G*power calculator was used to estimate a minimal sample size needed to perform a repeated measures ANOVA test between factors (*F-*test) using 2 groups, 2 measurements, and 0.8 correlation among measures as default for the A-priori estimation. Assuming an effect size of 0.50, 80% power, and a significance level of 0.05, a minimal sample size of 32 was needed for our primary outcome. Thus, a sample size of 46 was deemed adequate for the exploratory purpose of this pilot.

Multiple strategies were utilized to recruit and screen eligible participants, including 1) placing an advertisement on Craigslist, ResearchMatch, and University Listservs; 2) distributing recruitment flyers at rural primary care settings; and 3) applying snowball and word-of-mouth techniques with people who may have contacts (via Facebook) in rural communities. Our advertisements and flyers had a direct link (QR code) to our eligibility screening survey (Qualtrics) as well as a place for potential participants to leave their contact information or, if preferred, they could contact us directly with any questions. After that, one of our research assistants (RA) provided research briefing through a direct phone call in which we further verified their eligibility and interest. During this briefing, potential participants were informed that if enrolled they could be randomly assigned to a group that would either be receiving health coaching virtually or health information via email. Participants were informed that they would be contacted by another blinded research assistant to perform data collection to avoid potential bias. Once verbal consent was obtained, we mailed a digital weight scale and a growth wall chart to their homes. Once the package arrived, our RA made a zoom meeting (or phone call) to perform remote at-home anthropometric assessments to verify their eligibility (BMI 25-45). A Qualtrics survey and an automated self-administrated 24-hour (ASA24) dietary assessment tool (Bekelman et al., 2022) were then sent to each eligible participating parent. On the first page of the Qualtrics survey, participants were asked to proceed to the next page if they had given their consent to participate in the study. After receiving consent via the Qualtrics survey, we conducted the randomization process using a randomization table (stratified by participants’ weight category [overweight or obese]).

Since it is difficult to isolate participants from mindfulness practices or various lifestyle modifications, an active-control condition was applied to assess whether the control dyads could benefit from self-learning activities provided through emailed health information. Accordingly, the program title, ‘*Mindful-Healthy Family,’* appeared in all our communication materials (emails, letters, and envelopes) for both groups to sustain their mindfulness and motivation toward making lifestyle changes.

### Intervention and active-control groups

Intervention parents received nine MM-based-MI sessions (30‒45 minutes/each) every other week provided by extensively trained intervenors via Zoom (> 80%) or telephone calls (one-on-one per participant’s preference). MM-based-MI sessions utilized MI techniques and focused on exploring each parent’s unique perceived barriers/strengths, problem identification, motivators, and short  – and long-term goals. Table 1 outlines the content for each session. The principle of ‘*education with permission*’ was also applied to empower, elicit, and fulfill participants’ education needs (Kao et al., 2021a). In addition, effective mindfulness exercises and strategies obtained from our previous systematic reviews (Kao et al., 2023a; Kao et al., 2023b) were incorporated into this program (see Table 1 for details). To encourage parents’ practices at home, we provided intervention parents a copy of the workbook (or electronic copy if preferred). Intervention group parents also received mindful and motivational text messages three times a week during the intervention period to sustain their commitment.

To ensure treatment fidelity, all intervenors (nursing or medical students) were required to complete at least nine hours of MI and mindfulness training. Guided by the intervention manual, multiple role-plays were implemented and evaluated to ensure intervenors were adequately prepared to deliver the intervention accordingly. To minimize attrition, we sent a welcome and thank you card to participants after they were enrolled and allowed intervention parents to reschedule a session three times to accommodate unexpected events. All health coaching sessions were audio recorded and reviewed periodically to assess if there was a need for retraining. After completing each coaching session, the intervenor was required to complete a self-evaluation while another intervenor conducted a peer review after listening to the audio recording to ensure the completed session was delivered according to the intervention manual and congruent with MI and MM principles. Participants’ progression, such as their perceived importance, readiness, and confidence toward making lifestyle changes, as well as perceived barriers, strengths, and motivators were clearly documented. The goals of these assessments were to tailor intervention foci for the subsequent visits to increase participants’ self-efficacy and commitment toward making lifestyle changes.

Active-control parents received health education materials via email every other week for 18 weeks. Most of these materials were adapted from the Centers for Disease Control and Prevention (CDC) website. Health topics included COVID-19 prevention, benefits of vaccines, and links to the CDC homepage and other specific pages to share additional health information (e.g. tips on healthy eating, how to lower obesity-related health risks, weight management, and stress management).

### Data collection

One important goal of this pilot was to evaluate the feasibility of recruiting and engaging rural families remotely. All virtual contacts were clearly documented (or audio recorded) and tracked to evaluate our recruitment, assessment, intervention, and attainment efforts. Correspondingly, remote data collection was performed for all self-reported data (a Qualtrics survey and 24-hour recall of food consumption via ASA24) and physical assessments (dyads’ height and weight) at baseline and post-intervention (approximately 3‒5 months post baseline assessment). A Qualtrics survey with informed consent was sent to participants via email or text (if preferred) for completion at baseline. Moreover, parents’ dietary intake data (24-hour recall or food records) were collected and analyzed using the ASA24 dietary assessment tool developed by the National Cancer Institute (Bekelman et al., 2022). For the remote anthropometric assessment, we used incentive materials delivered to their home, which included a bathroom weight scale (*Triomph Precision Body Fat scale)*, and a growth wall chart (*Home Wall Décor Height Indicator*). To ensure the accuracy of these assessments, we provided paper instructions three days in advance on how to use these tools to obtain their height and weight. During the Zoom meetings (video preferred), participants were instructed to measure their height and weight twice to the nearest 0.1 cm and 0.01 kg, respectively. If, for any reason, the video was not viable, we asked participants to take and send pictures of their measurements to us for verification.

### Measures (feasibility, acceptability, and preliminary effects)

**Feasibility** of enrollment was evaluated by the number of potential participants reached in each step outlined in the CONSORT flow chart. The attendance rate was calculated by assessing the number of doses delivered divided by the total number of doses intended (x 100%). The attrition rate was calculated using cases that did not complete the post-assessment divided by the total enrolled N (x 100%). Moreover, adopted from the MITI coding manual and MM principles, a Progression Evaluation Form and Coding Sheet were developed to assess treatment fidelity (see Supplemental Table 1). This 12-item 5-point scale coding sheet was designed for paired intervenors to assess the adherence to MI principles (5 items) and MM principles (7 items) after listening to each session recording. A Progression Evaluation Form was designed for intervenors to reflect and document the content of each health coaching session.

**Acceptability** of the *Mindful Healthy Family* program was evaluated with 11 questions (developed by the study team) asking intervention parents to rate their perceptions (1‒5 scale, from ‘*not at all’* to ‘*every much’*) with various aspects of the intervention at post-intervention. In addition, we used four open-ended questions to elicit parents’ perceptions about the program’s strength, areas for improvement, cultural relevance, and tips to enhance participants’ commitment (Supplemental Table 2). Participants’ acceptability (satisfaction) was calculated using the proportion of rating that showed a satisfactory assessment (≥3 out of 5 points).

**Preliminary effects** were assessed using the following measures, which included a demographic survey developed by the study team to determine participants’ characteristics. This survey included questions on dyads’ ages, sex, ethnicity, race, family annual income, parental marital status, employment status, and education level.

**Parents’ dietary intake** was assessed using the Health Eating Index (HEI-2015). To calculate HEI scores for personal level ASA24 dietary intake, we used the HEI SAS code available on the HEI webpages (Bekelman et al., 2022). In this pilot, we focused on parents’ HEI dietary intake, particularly with regard to components including HEI-total score (ranging from 1‒100, an ideal score for adults is 100), total vegetable intake, intake of greens and beans, saturated fat, total protein, added sugars, and sodium (Krebs-Smith et al., 2018). Children’s dietary intake in the past week was assessed using the Nutrition Quest Food Frequency Questionnaire (FFQ) and the Block Kids Food Screener for ages 2‒17 years (Hunsberger et al., 2015). Special focuses were on total calories (Kcal), fiber, fat, protein, and sugar added. A higher sum score indicates higher consumption in each category.

Additional secondary outcomes included parents’ PA, mindfulness eating, and exercise self-efficacy, as well as children’s PA. The International Physical Activity Questionnaire (IPAQ)-short form was used to assess parents’ metabolic equivalent of task (MET), which is the sum of walking, moderate, and vigorous MET-minutes/week (Booth, 2000). The 28-item Mindfulness Eating Questionnaire (MEQ) (Framson et al., 2009) and 9-item Exercise Self-Efficacy Scale (Resnick & Jenkins, 2000) were used to assess individuals’ mindfulness eating and their self-efficacy for exercise. A higher score represents a higher level of self-efficacy in exercise. All the above selected measures have been validated and have adequate reliability. The reliability of MEQ and exercise self-efficacy in this pilot was adequate (ranging from .79 to .94). Children’s PA was evaluated using a 1-item survey that asked parents to estimate the number of days in the past 7 days that their child had spent at least 60 minutes engaging in PA per day (0‒7 days).

**BMI** was calculated using a digital bathroom weight scale (*Triomph Precision Body Fat scale)* and a growth wall chart (*Home Wall Décor Height Indicator*). If the two measurements differed by ≥0.5 cm for height or ≥0.5 kg for weight, a third measurement was executed and the two closest measurements were averaged to determine the final measurment value. Children’s BMI for age and sex was determined using growth charts available online from the CDC (Centers for Disease Control and Prevention, 2022).

Parents’ depressive symptoms and stress were assessed using the 10-item Center for Epidemiological Studies Depression Scale (CES-D) (Irwin et al., 1999) and 10-item Perceived Stress Scale (Cohen et al., 1983). Both validated measures have been widely used with good reliabilities (ranging from .71 to .91). Our reliability is slightly lower than the original ones (ranging from .63 to .85) obtained from the pre to post assessments. Higher scores indicate more depressive symptoms or a higher level of perceived stress.

**Family satisfaction and collective efficacy** were assessed using the 5-item Family Adaptation, Partnership, Growth, Affection, and Resolve (APGAR) scale(Smilkstein, 1978) and the 20-item Perceived Collective Family Efficacy scale (Caprara et al., 2004), respectively. Both measures have excellent validity and reliability (ICC ranging from 0.96–0.98 and Cronbach’s alpha  = 0.92, respectively) and have been broadly used to reflect parents’ satisfaction and collective efficacy within the family context. Our reliability was similar (Cronbach’s alpha ranging from .79 to .93 for both assessments). Higher scores represent higher levels of family satisfaction or collective efficacy.

### Data analysis

All data analyses were conducted using IBM SPSS Statistics 28. Intention-to-treat principle was applied for all analyses. Study variables were described by means, standard deviations, frequencies, and percentages. To assess the demographic differences between groups at baseline, independent t-tests were applied for continuous variables and Chi-square (*χ^2^)* tests were conducted for categorical variables. The patterns of missing data were displayed, tabulated, and found to be at random (Little’s MCAR test, *χ^2 ^*= 148.69*, df *= 125*, p *= .073*)*. To examine intervention effects, two-way mixed ANOVA was applied (time as the within subject factor = 2 using the pre and post values; group as the between group factor) while controlling for parents’ marital status (as covariates), because marital status differed significantly between the groups (*χ^2 ^= * 8.214; *p* = .016). We first examined the potential interaction effect (group*time) on the dependent variable. When the interaction effects were not significant, we then assessed the between-group and within-group (time) effects separately. Following the same procedures, additional adjustments (child’s age and sex) were applied when assessing intervention effects on children’s outcomes. Adjusted effect sizes of η^2^ value were used to describe the between-group intervention effects: small (η^2^ = 0.01), medium (η^2^** **= 0.06), and large (η^2^ = 0.14). A conversion from η^2^ to Cohen’s *d* was calculated for easy interpretation: small (*d *= 0.2), medium (*d *= 0.5), and large (*d *= 0.8) (Fritz et al., 2012). Positive effect size *d* indicates that the intervention group had better effects (a greater increase) than the active-control group, while negative effect size shows that the active-control group’s increase overperformed the intervention group on the outcome. Statistical significance was indicated by the *p*-value (≤.05). Because this study was designed for the purpose of conducting a preliminary analysis(Indrayan & Mishra, 2021) in which the goal was to explore and generate the effect sizes for future confirmatory clinical trials (power estimation), multiple testing corrections to control for Type 1 errors were not performed.

## Results

### Participants

Table 2 shows participants’ demographic characteristics. Most participants’ racial background was white (n = 33, 72%) followed by Hispanic (n = 5, 11%), mixed race (n = 5, 11%), black (n = 4, 9%), and Native American (n = 4, 9%). The demographic characteristics were comparable between the intervention and active-control group at baseline except for their marital status (*p *= .016), in which intervention parents were less likely to be married or living with a partner.

**Table 2.** Family Demographics at Baseline.

Continuous Variables	Total (N = 46)		Control (n = 17)		Intervention (n = 29)		Difference	
							t-test	p
Parent age (mean ± SD)	38.35 ± 5.		39.61 ± 5.81		37.54 ± 4.74		1.326	.462
Child age (mean ± SD)	6.90 ± 2.61		7.58 ± 1.99		6.46 ± 2.89		1.4338	.102
Parent BMI (mean ± SD)	32.69 ± 6.08		31.38 ± 5.09		33.53 ± 6.59		−1.173	.082
Child BMI (mean ± SD)	17.94 ± 5.06		18.19 ± 6.16		17.79 ± 4.33		0.258	.727
Categorical Variables	Total		Control		Intervention		Group difference	
	N	%	N	%	N	*%*	*X^2^*	*p*
Parent sex: Female	39	84.8	16	94.1	23	79.3	17.83	.400
Child sex: Female	26	56.5	11	64.7	15	51.7	1.238	.266
**Parents**								
Ethnicity:						* *	* *	
Hispanic	4	8.7	2	11.8	2	*6*.*9*	*1*.*279*	.258
Race						* *	3.666	.300
White	36	78.3	14	82.4	22	75.9		
Black	3	6.5	2	11.8	1	3.4		
Native American	4	8.7	0	0	4	13.8		
Mixed	3	6.5	1	5.9	2	6.9		
Employment: Full-time	34	73.9	13	76.5	21	72.4	0.394	.821
Part-time	6	13.0	3	17.6	3	10.3		
No	6	13.0	1	5.9	5	17.2		
Education							* *2.850	.583
≤High school	3	6.5	0	0	3	10.3		
Some college	15	32.6	5	29.4	10	34.5		
4-year college degree	10	21.7	5	29.4	5	17.2		
Professional degree	18	39.1	7	41.2	11	37.9		
Annual income							3.398	.494
≤29K	7	15.2	1	5.9	6	20.7		
$30 K to 49K	7	15.2	2	11.8	5	17.2		
$50 K to 99 K	17	37.0	8	47.1	9	37%		
≥$10K	15	32.6	6	35.3	9	31.0		
Marital Status							**8.214**	**.016**
Married, partnership	36	78.3	17	100	19	65.5		
Separated, divorced, widowed	3	6.5	0	0	3	10.3		
Single	7	15.2	0	0	7	24.1		

Note. **Bold** font indicates a statistically significant difference between groups (*p *< .05).

### Feasibility and treatment fidelity

**Figure 1.**
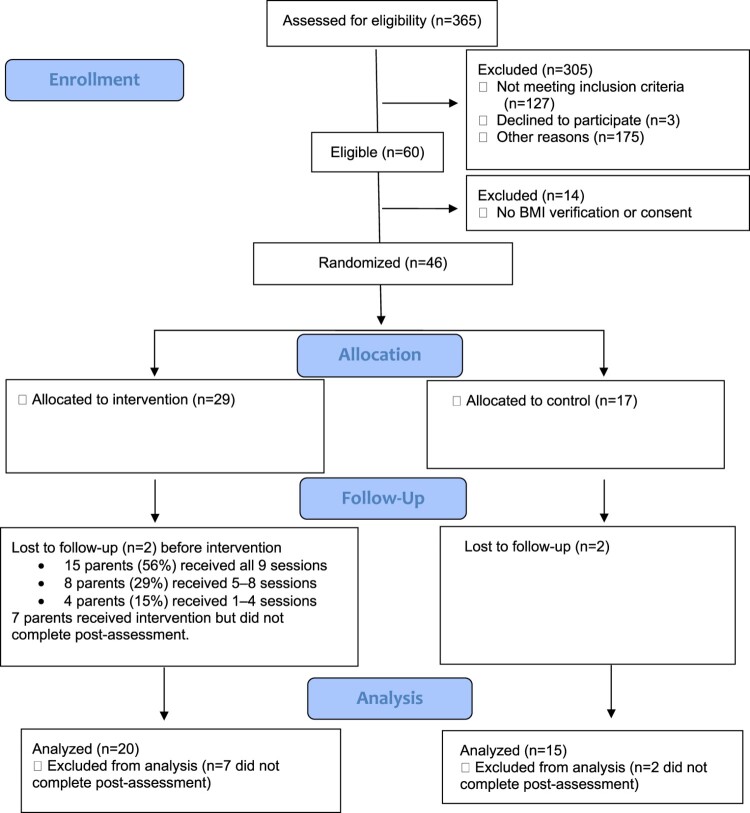
CONSORT Flow Diagram.

Figure 1 illustrates the study’s flow diagram. Among the 365 individuals who completed eligibility screening, 305 (83.4%) were excluded for various reasons. Of the 60 eligible participants, 14 did not complete either the formal consent or remote anthropometric verification after receiving the incentive material package and subsequently were dropped from participation. In the end, 46 parent–child dyads were enrolled and randomized (stratified by parents’ weight category [overweight or obese]). Among 29 families allocated to the intervention group, two dropped out of the study before receiving the intervention. For the remaining 27 intervention parents, 15 parents (56%) received all nine sessions, while 8 (25%) received 5‒8 sessions, and 4 (14%) received 1‒4 sessions. In total, 194 sessions (out of 243) were delivered with an attendance rate of 79.8%. All intended health education handouts were delivered to active-control group parents via email without any rejections. Of the 46 enrolled dyads, 35 completed post-intervention assessments for a 23.9% attrition rate. Treatment fidelity was confirmed by reviewing 194 session recordings/transcriptions. The results indicated that all trained intervenors had generally adhered to the MI and MM principles and intervention manual. The mean score of peer review coding was 4.13 (1 = low and 5 = high adherence). Participants’ perceived importance, readiness, and confidence were also enhanced over time, particularly for those who completed at least five sessions per intervenors’ post evaluation. Detailed implementation and process evaluations with additional qualitative data will be reported elsewhere.

### Acceptability

Overall, intervention parents reported significantly higher program satisfaction than the active-control parents (*p* < .05). The virtual one-on-one health coaching sessions were favored/endorsed by all intervention parents. More than 92% of intervention parents reported favorable satisfactory responses (Supplemental Table 2). Regardless, parents’ frequent requests to reschedule sessions was reported as being of great concern for intervenors who sought to complete the intervention within the projected timeframe. Finally, most participating parents (≥90%) welcomed and frequently used the weight scale and growth chart sent to their homes and felt confident about measuring their height and weight.

### Intervention effects (between-group effect)

**Figure 2.**
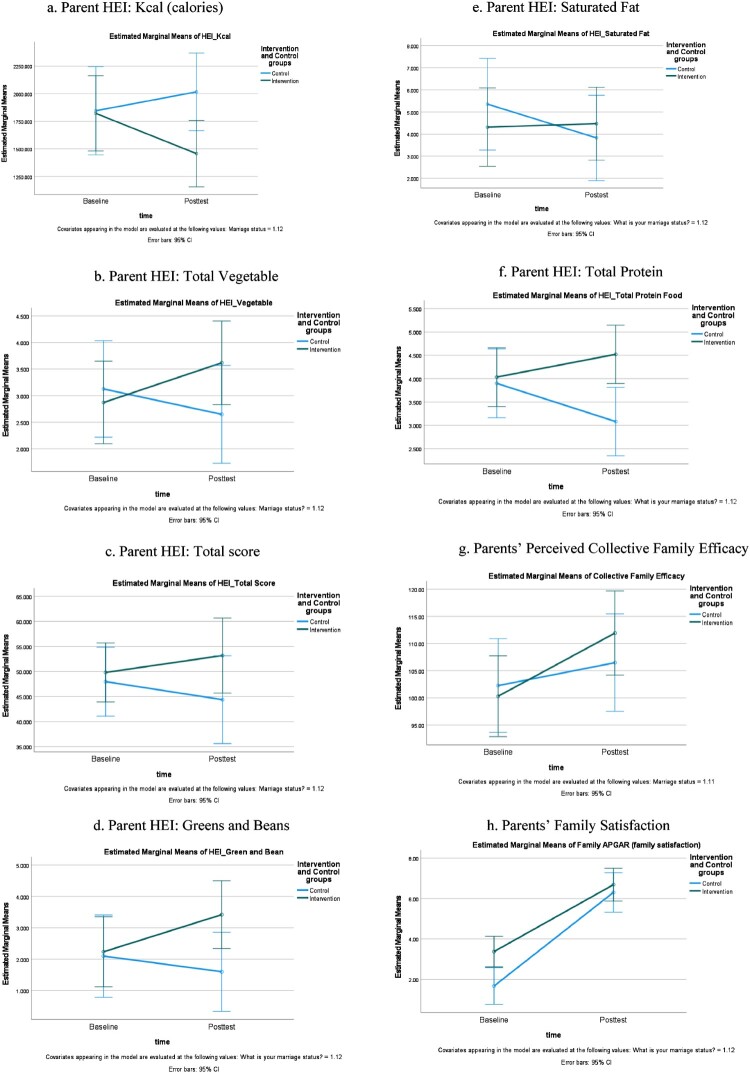
Parent HEI and Family Function.

**Participating Parents:** No significant interaction effects were noted on all targeted outcomes. Table 3 provides both between-and within-group effects. Although not statistically significant, the intervention parents had small effects on ↑HEI total score (η^2 ^= 0.04, *d *= 0.43; Figure 2c), ↑total vegetable (η^2 ^= 0.04, *d *= 0.41; Figure 2b), ↓depressive symptoms (η^2 ^= 0.04; *d *= −0.42) and ↑exercise self-efficacy (η^2 ^= 0.01, *d *= 0.21) while controlling for marital status. Similarly, medium effects on ↓HEI Kcal (η^2 ^= 0.08, *d *= −0.58; Figure 2a), ↓added sugar (η^2 ^= 0.06, *d *= −0.50), and ↑total greens/beans (η^2 ^= 0.08; *d *= 0.59; Figure 2d) were also observed. Moreover, a large effect was observed for ↑total protein intake (η^2 ^= 0.14; *d *= 0.82). In terms of family satisfaction, we found a medium effect (η^2 ^= 0.06; *d *= −0.50) that favored the active-control parents. However, it is essential to note that control parents’ family satisfaction was significantly lower than that of the intervention parents at baseline (*F_1,43 =_* 4.054, *p *= 0.05; Figure 2h.).

**Table 3.** Intervention Effects (between-group and within group).

Outcomes	Control group			Intervention group			Between-Group effect size	
	Pre mean ± SD (n = 15)	Post mean ± SD (n = 15)	Within-group effect	Pre mean ± SD (n = 20)	Post mean ± SD (n = 20)	Within-group effect	η^2^	*d_cohen_*
			***d_cohen_*** (95%CI)			***d_cohen_*** (95%CI)		
**Primary Outcomes (Parent Dietary Intake [ASA24-2015])**								** **
HEI_Total Score	47.40 ± 15.02	45.82 ± 14.68	−0.11(−1.155–0.942)	50.24 ± 9.93	52.13 ± 17.25	0.13(−0.766–1.035)	**0**.**04^a^**	**0**.**43^a^**
HEI_Kcal	1844.61 ± 837.93	2017.18 ± 667.24	**0.23^a^**(−0.823 - 1.279)	1830.23 ± 605.77	1456.37 ± 602.64	**−0.62**(−1.565–0.327)	**0**.**08^b^**	**−0**.**58^a^**
HEI_Total Vegetable	2.97 ± 1.66	2.69 ± 1.51	−0.18(−1.226–0.873)	2.99 ± 1.72	3.59 ± 1.72	**0.35^a^**(−0.557 - 1.255)	**0**.**04^a^**	**0**.**41^a^**
HEI_Green and Bean	2.08 ± 2.33	1.78 ± 2.26	−0.13(−0.872–0.611)	2.25 ± 2.31	3.29 ± 2.35	**0.45^a^**(−0.197–1.09)	**0**.**08^b^**	**0**.**59^a^**
HEI_Saturated Fat	4.87 ± 3.95	3.58 ± 3.99	**−0.33^a^**(−1.071–0.421)	4.67 ± 4.04(n = 19)	4.66 ± 3.13(n = 19)	−0.003(−0.639–0.633)	0.00	0.06
HEI_Total Protein	3.87 ± 1.38	3.25 ± 1.67	**−0.41^a^**(−1.153–0.344)	4.05 ± 1.24	4.40 ± 1.20	**0.29^a^**(−0.352–0.926)	**0**.**14^c^**	**0**.**82**
HEI_Added Sugar	6.63 ± 3.48	7.28 ± 2.94	**0.20^a^**(−0.541–0.944)	7.67 ± 2.81	7.88 ± 1.76	0.09(−0.547–0.726)	**0**.**06^b^**	**−0**.**50^b^**
HEI_Sodium	4.00 ± 3.57(n = 14)	3.67 ± 3.33(n = 14)	−0.096(−0.837–0.646)	3.51 ± 2.87 (n = 19)	3.73 ± 3.29 (n = 19)	0.07(−0.565–0.707)	0.00	0.06
**Secondary Outcome (Parents)**								** **
Mindful Eating Score	71.87 ± 11.39	76.07 ± 11.39	**0.37^a^**(−0.652- 1.389)	73.80 ± 9.65	78.70 ± 12.45	**0.44^a^**(−0.447–1.327)	0.01	0.18
Collective Family Efficacy	104.0 ± 15.36	107.47 ± 14.65	**0.23^a^**(−0.784 −1.247)	99.0 ± 18.04	111.2 ± 18.30	**0.67^b^**(0.03–1.57)	0.00	0.13
Perceived Stress	24.21 ± 5.44	21.07 ± 7.38	**−0.48^a^**(−1.547–0.579)	24.30 ± 5.50	21.85 ± 5.12	**−0.46^a^**(−1.349–0.427)	0.001	0.06
^¥^Family Satisfaction	1.64 ± 1.74	6.21 ± 1.53	**2.79^c^(1.749-3.83)**	3.40 ± 1.54	6.75 ± 1.89	**1.94^c^(1.191-2.695)**	**0**.**06^a^**	**−0**.**50^a^**
Depressive Symptom	7.84 ± 3.98	7.54 ± 5.28	−0.06(−0.833–0.705)	6.94 ± 4.31	6.42 ± 5.31	−0.11(−0.744–0.529)	**0**.**04^a^**	**−0**.**42^a^**
Self-Efficacy in Exercise	36.8 ± 26.52	39.53 ± 30.55	0.10(−0.621- 0.812)	38.15 ± 22.95	43.8 ± 18.21	**0.27^a^**(−0.35–0.895)	**0**.**01^a^**	**0**.**21^a^**
PA_MET	2088.0 ± 1984.8	3539.3 ± 3488.6	**0.51**^b^(−0.517–1.54)	2491.8 ± 2492.6	1973.7 ± 1586.2	**−0.25^a^**(−1.128–0.632)	0.01	−0.18
BMI	30.52 ± 5.30	30.37 ± 5.16	– 0.03(−1.116–1.059)	32.14 ± 6.10	32.08 ± 6.17	−0.01(−1.097–1.077)	0.003	0.11
**Secondary Outcomes (Children)** η^2^ value was obtained after adjusted for child’s age and sex as well as parental marital status								** **
Block Screener Kcal	1764.64 ± 1292.84	1188.11 ± 290.05	**−0.62^b^**(−1.825–0.594)	1109.66 ± 342.98	1015.47 ± 297.20	**−0.29**^a^ (−1.249–0.662)	**0**.**09^b^**	**0**.**63^b^**
^¥^Fiber Intake	15.14 ± 9.83	11.24 ± 3.50	**−0.53^b^**(−1.731–0.674)	8.81 ± 3.56	8.93 ± 3.94	0.03(−0.919–0.983)	**0**.**12^b^**	**0**.**75^b^**
Total Fat	74.17 ± 54.58	49.67 ± 13.54	**−0.62^b^**(−1.826–0.594)	47.95 ± 14.32	42.85 ± 14.06	**−0.36^a^**(−1.318–0.599)	**0**.**09^b^**	**0**.**66^b^**
^¥^Total Protein	77.50 ± 51.33	50.36 ± 17.30	**−0.71^b^**(−1.927–0.51)	47.14 ± 14.98	44.84 ± 16.26	−0.15(−1.099–0.805)	**0**.**11^b^**	**0.72 ^b^**
Sugar Added	10.13 ± 14.13	6.12 ± 5.18	−**0.38^a^**(−1.569–0.816)	6.42 ± 4.01	5.29 ± 2.39	**−0.34^a^**(−1.3–0.615)	0.004	0.13
BMI percentile	64.27 ± 31.95	63.55 ± 31.74	−0.023 (−1.205–1.159)	64.53 ± 33.44	68.97 ± 31.17	0.14(−0.876–1.151)	0.000	0.000
Child PA(0‒7 days)	5.27 ± 1.58	5.93 ± 1.49	**0.43^a^** (−0.594–1.454)	5.40 ± 1.79	4.75 ± 2.49	**−0.30^a^**(−1.181–0.582)	**0**.**05^a^**	**−0**.**48^a^**

*p < 0.05; **η^2^** value was obtained from two-way mixed ANOVA analysis while controlling for marital status; **η^2^** = 0.01, small effect; **η^2^** = 0.06, medium effect; **η^2^ **= 0.14, large effect; *d_Cohn_*  = 0.2, small effect; *d_Cohn_*  = 0.5, medium effect; *d_Cohn_  *= 0.8, large effect. **^a^** = small effect size; **^b^** = medium effect size; **^c ^**= large effect size. Bold **^a, b, c^** indicates effect sizes ≥ the recommended cut point of **η^2^**, **d_cohen_**; 95%CI = 95% confidence interval. ^¥^ Significantly different baseline values between groups.

**Participating Children**: Medium intervention effects were found on ↑fiber (η^2 ^= 0.12; *d *= 0.75; Supplemental Figure S1.f.), and ↑protein (η^2 ^= 0.11; *d *= 0.72; Figure S1.h.) when controlling for children’s age, sex, and parents’ marital status. However, the active-control group showed better effects on decreasing children’s Kcal (η^2 ^= 0.09, *d *= .63; Figure S1.e.), total fat (η^2 ^= 0.10; *d *= 0.66; Figure S1.g.) and increasing PA (η^2 ^= 0.05, *d *= −0.48; Figure S1.d.). Specifically, intervention children’s decrease in Kcal was smaller than for the children in the control group. Moreover, congruent with parents’ PA, control group children’s PA days increased while intervention children had a decrease in PA days.

### Outcome changes over time (within-group effects)

**Participating Parents**: Table 2 provides the outcome variables, sample size, mean, and standard deviation (SD) obtained at baseline and post-intervention by group. Among the intervention parents, the MM-based-MI had small effects on ↑vegetable (*d *= 0.34, 95%CI: – 0.557–1.255), ↑total green/bean (*d *= 0.45, 95%CI: – 0.197–1.09), ↑protein (*d *= 0.29, 95%CI: – 0.325–0.926), ↑mindful eating score (*d *= 0.44, 95%CI: – 0.447–1.327), ↑exercise self-efficacy (*d *= 0.27, 95%CI: – 0.350–0.895), ↓PA_MET (*d *= −0.25, 95%CI: – 1.097–1.077), and ↓perceived stress (*d *= −0.46, 95%CI: – 1.349 0.427). We found medium effects on ↓HEI Kcal (*d *= −0.62, 95%CI: – 1.565–0.327) and ↑collective family efficacy (*d *= 0.67, 95%CI: 0.03–1.57) as well as a large effect on ↑family satisfaction (*d *= 1.94, 95%CI: 1.191–2.695) for intervention parents.

For the active-control group, small effects were noted on ↑Kcal (*d *= 0.23, 95%CI: – 0.823–1.279), ↓saturated fat (*d *= −0.33, 95%CI: – 1.071–0.421), ↓protein (*d *= −0.41, 95%CI: – 1.153–0.344), ↑added sugar (*d *= 0.20, 95%CI: – 0.541– 0.944), ↑mindful eating (*d *= 0.37, 95%CI: – 0.654–1.389), ↑collective family efficacy (*d *= 0.23, 95%CI: – 0.784–1.247), and ↓perceived stress (*d *= −0.48, 95%CI: – 1.547–0.579). In addition, we found a large effect on ↑family satisfaction (*d *= 2.79, 95%CI: 1.749–3.83) and a medium effect on ↑PA-MET (*d *= 0.51, 95%CI: – 0.517–1.54) among active-control parents.

**Participating Children:** Children’s dietary intake was improved over time. For the intervention group children, small within-group effects were noted on ↓Kcal (*d *= −0.294, 95%CI: – 1.249–0.662), ↓total fat (*d *= −0.359, 95%CI: – 1.318–0.599), ↓sugar added (*d *= −0.342, 95%CI: – 1.3–0.615), and ↓PA days (*d *= −0.3, 95%CI: – 1.181–0.582). For control group children, small within-group effects were noted on ↓sugar added (*d *= −0.377, 95%CI: – 1.569–0.819) and ↑PA days (*d *= 0.43, 95%CI: – 0.594–1.454). In addition, medium effects were noted on ↓Kcal (*d *= −0.615, 95%CI: – 1.825–0.594), ↓fiber intake (*d *= −0.529, 95%CI: – 1.731–0.674), ↓total fat (*d *= −0.616, 95%CI: – 1.826–0.594), and ↓protein (*d *= −0.709, 95%CI: – 1.927–0.510; Figure S1.h.). It is essential to note that control group children had significantly higher fiber intake (*F_1, 26_* = 5.945, *p *= 0.022) and total protein (*F_1, 26_* = 5.345, *p *= 0.029) at baseline compared to intervention group children.

## Discussion

The goals of this RCT were to examine the feasibility, acceptability, and preliminary effects of a fully virtual MM-based-MI program on parents’ Healthy Eating Index (HEI), collective family efficacy, family satisfaction, perceived stress, and depressive symptoms as well as the parent–child dyads’ BMI, eating, and physical activity (PA). This trial was pragmatic in nature and focused more on external validity to increase the application of mindfulness-based lifestyle modifications in a real-world setting to prevent obesogenic behaviors (Patsopoulos, 2011). Although further investigation with a larger sample size is warranted, the positive findings of this pilot indicate that delivering mindfulness-based intervention virtually using MI techniques is feasible and was well-accepted by rural parents. The preliminary effects were very promising despite the negative influence of the COVID-19 pandemic on obesogenic behaviors (Almandoz et al., 2020; Jia, 2021).

The feasibility of this virtural pilot was clearly reflected by having relatively good enrollment, attendance, and attrition rates. Although our retention and attendance rates were considered adequate (Amico, 2009) and good (Tuda et al., 2022) respectfully for a healthy lifestyle program among rural parent–child dyads, improvements are needed to enhance our enrollment rate (Bieganek et al., 2022). In addition, our attention and evaluation to treatment fidelity further informed that our virtual program was implemented as intended and consistently throughout the study. Finally, the acceptance of this pilot was obviously endorsed by the great satisfaction (>92%) reported by the intervention parents.

The MM-based-MI intervention exhibited very encouraging small to large positive effects on parents’ HEI in terms of total score, total vegetable intake, added sugar, calories, and total protein compared to the active-control group, after controlling for marital status. The positive between-group effects on rural parents’ dietary intake (primary outcome) were very encouraging because of the critical need to provide obesity prevention services to rural families who are at increased risk for being overweight (Fanzo, 2018). In fact, rural families are faced with increasing challenges derived from limited natural resources, climate change, and suburbanization. Development of effective and accessible strategies to improve rural parents’ dietary practices may serve as an essential first step in overcoming these environmental challenges.

Of the secondary outcomes, after controlling for marital status, the positive small intervention effects on improving family satisfaction, depressive symptoms, and exercise self-efficacy were also meaningful because people’s emotional state can affect their ability to engage in everyday activities as well as their overall mental and physical health. Compared to active-control parents, intervention parents’ improvements in emotional well-being and exercise self-efficacy may play a significant role in their adoption of healthy lifestyles (Hendriks et al., 2017). Moreover, we observed increases on mindful eating, collective family efficacy, and family satisfaction in both groups. This result may be rooted in participants’ enthusiasm and motivation to proactively seek ways to enhance their healthy lifestyle behaviors and family function. Despite this therapeutic value found in both groups, it is important to note that intervention parents’ increase in collective family efficacy was much greater than for active-control parents (0.67 vs. 0.23). This increase may be derived from our successful ‘*mindful interaction’* component, which included positive mindful parenting strategies and various trainings focused on compassion (for self and others). The rise in collective family efficacy is fundamental and consistent with the components of the Family Resilience Model because it can significantly buffer both parents and children from engaging in risky health behaviors (Kao et al., 2020; Kao et al., 2021b).

The relatively small effect sizes on dyads’ BMI were expected because this MM-based-MI program is not intended for weight reduction. Rather, this pilot was designed to improve parent–child dyads’ dietary intake, emotional well-being, and family functioning. Moreover, when using the IPAQ-SF to assess parents’ PA, active-control parents had a greater increase in metabolic equivalent of task (MET) than intervention group parents. This unanticipated finding may be related to the IPAQ-SF measure’s lack of capacity to capture the mindful movements (e.g. yoga and mindful slower walking) that we had taught regarding mindful movement activities. Thus, for future programs, it might be important to incorporate some moderate and vigorous activities as part of the mindful movement curriculum. However, the increase in PA for control parents may reflect their attempts to lead a healthier lifestyle (being more active). Although both groups showed increased family satisfaction, the increase was significantly higher for control parents compared to intervention parents. This unexpected finding may be related to demographic differences between the groups. In particular, all control parents were married and had a relatively higher education level and annual income; therefore, they might have a higher capability for self-directed learning and achieve a greater increase in family satisfaction. In addition, the control parents had a much lower family satisfaction score at baseline. These factors may explain the different group effects in parents’ perceived family satisfaction.

Although we did not provide any direct interventions to the children, the improved dietary intake among children in both groups may be derived from the parents’ strong determination to lead a healthier lifestyle and create a healthier environment not only for themselves but also for their children. This positive influence is congruent with the assumptions of the Social Ecological Model, which suggests that the family environment can influence family members’ health status and emotional well-being (McCormick et al., 2021). The small to medium effects on children’s dietary intake and PA days also were consistent with the changes found in parents. It is possible that parental and family lifestyle changes can be diffused to the children. Thus, our vision for parents serving as change agents for their family members to lead healthier lifestyles is viable.

## Strengths and limitations

There are several limitations worth mentioning. First, with a small sample size, the power to generate significant findings is very limited (observed power ranged from 11% to 63%). Since we did not perform the multiple testing adjustments, caution should be applied when interpreting our results mainly due to the potential for Type 1 errors (false positive). This study should be replicated with larger trials with sufficient power to verify the positive intervention effects for rural parent–child dyads. Second, the time to complete this pilot was longer than what was initially anticipated. The delay was mostly related to protocol changes in order to make it a 100% virtual program to align with the global pandemic context and the many re-trainings that were required to implement this change. A more in-depth implementation and process evaluation should be conducted to further assess the strength and weakness of this pilot. Third, with self-reported measures, reporting bias may be present. Fourth, although all parents were confident about assessing their own weight and height, measurement errors may still be present. Fifth, we have named our comparison group as an active-control group because we had little control over the extent of learning that may have occurred or been triggered by our educational materials delivered by emails. Because we stressed ‘mindfulness’ and ‘healthy lifestyles’ in all our recruitment and communication materials, a certain level of exposure (contamination) to mindfulness practices was expected. However, it is also possible that some control parents may not have read the educational material at all. Regardless, using the term ‘active-control’ group demarcated that group from a placebo-controlled design.

Despite these abovementioned limitations, several strengths should be highlighted. First, our results support the promise of using fully virtual MM-based-MI interventions to improve and strengthen healthy lifestyles, emotional well-being, and family functioning among rural families. Second, our preliminary program evaluation revealed that this virtual pilot was well-accepted by the rural parents who reported great satisfaction (>92%) with the intervention. Third, the promising preliminary effects on mitigating obesogenic behaviors among rural families would be helpful to determine adequate sample size for future studies. Finally, our program focused on encouraging rural parents to act as family change agents, which holds promise for preventing and reducing obesogenic behaviors within the family context.

## Supplementary Material

MHF_supplementals_REV_CLEAN.docx

## Data Availability

Data not available: The participants of this study did not give written consent for their data to be shared publicly.
