# Supplementary material for: Feasibility and preliminary effects of the mindful healthy family project among rural families
Source: Health Psychol Behav Med. 2024 Dec 26;13(1):2446368. doi: 10.1080/21642850.2024.2446368 (PMC11703050; doi:10.1080/21642850.2024.2446368)
Supplement: MHF_supplementals_REV_CLEAN.docx [file RHPB_A_2446368_SM0521.docx]

**Supplemental Figure 1: Parent Perceived Stress, Self-Efficacy, PA, and Child Dietary Intake**

S1.a. Parents’ Depressive Symptoms


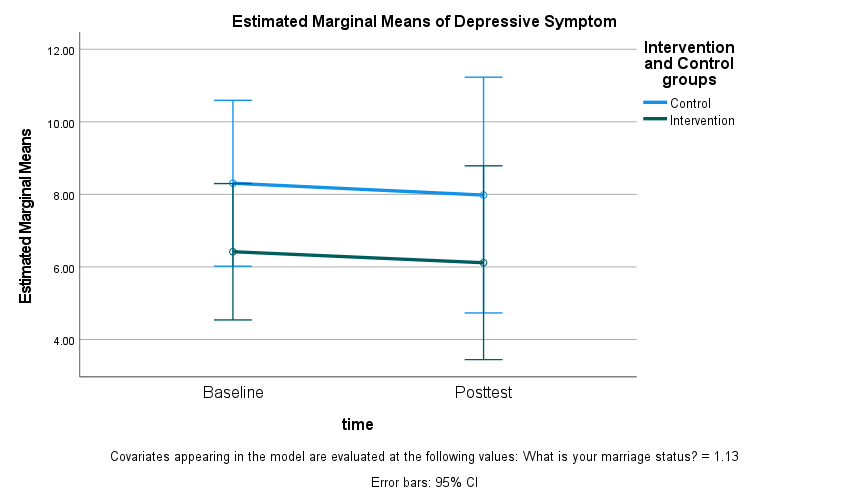


S1.b. Parents’ Exercise Self-Efficacy


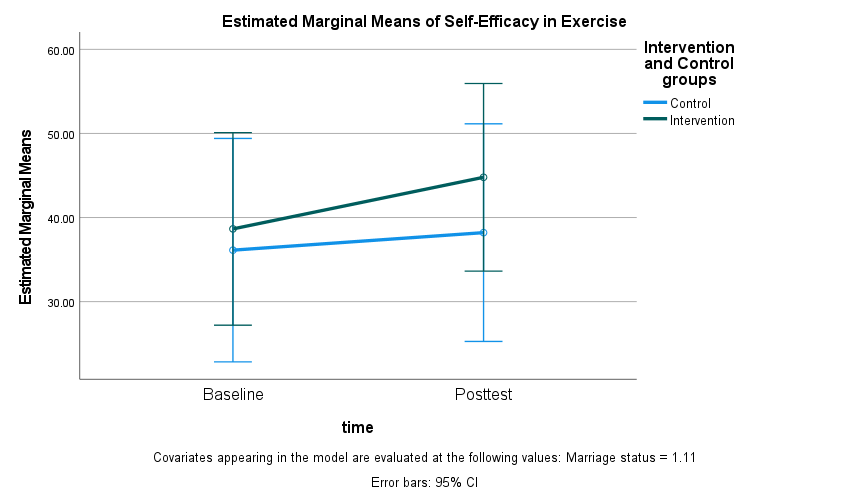


S1.c. Parents’ Physical Activity (MET-min)


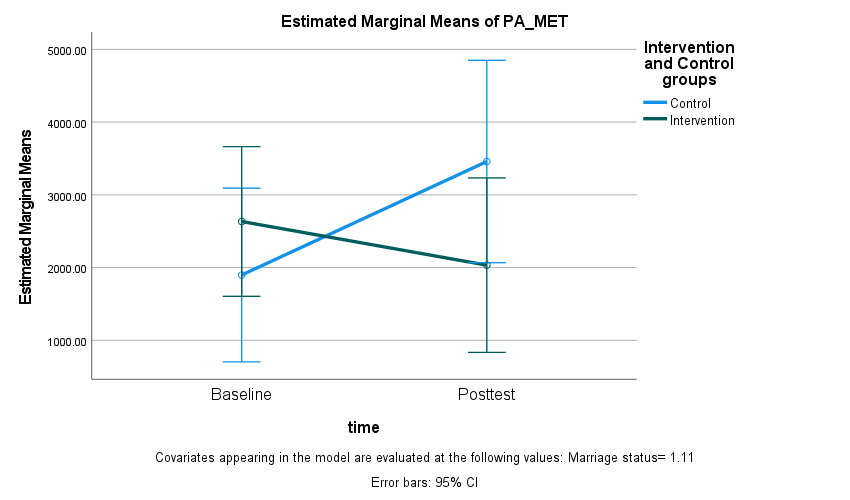


S1.d. Children’s PA (0‒7 days)


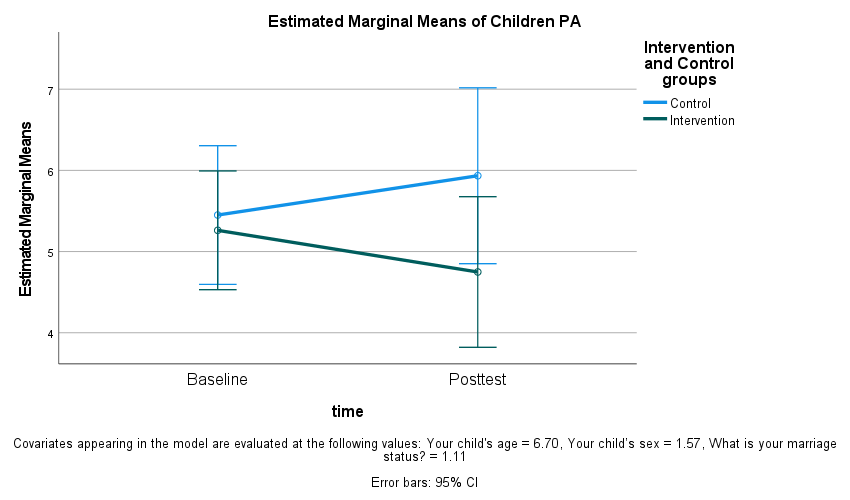


**Child Dietary Intake**

S1.e. Children’s Kcal (calories)


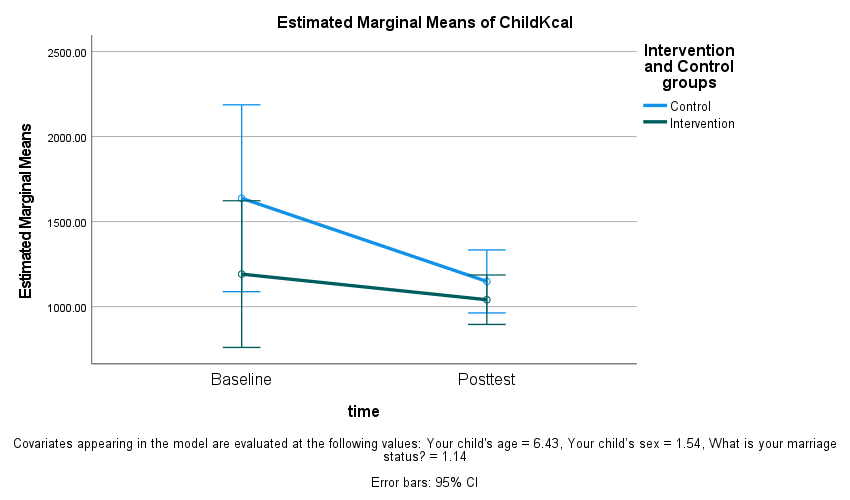


S1.f. Children’s Fiber Intake


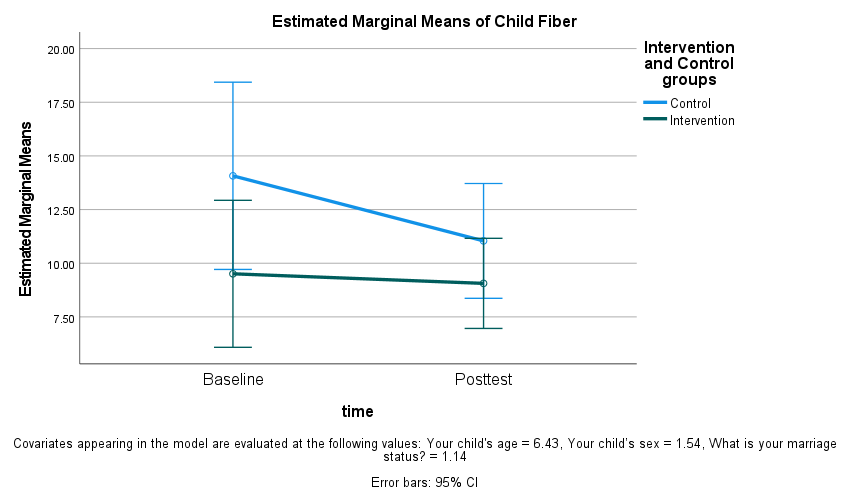


S1.g. Children’s Total Fat


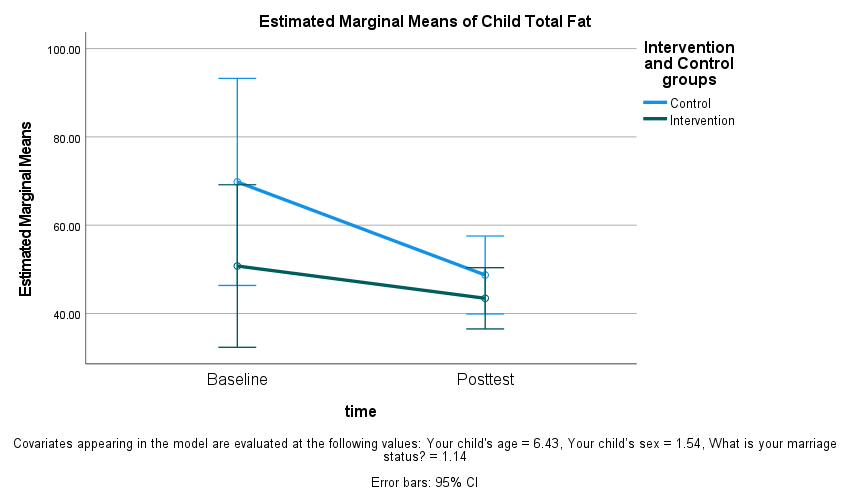


S1.h. Children’s Total Protein


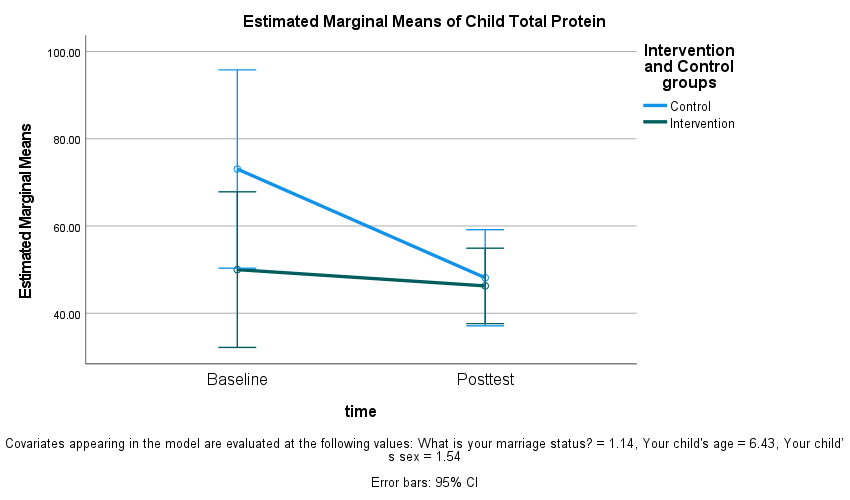


**Alt Text for Supplemental Figure 1** (53 words)

Eight separate diagrams (line drawings) illustrated the changes noted from baseline to post-intervention between the parents (or children) allocated in the intervention and active-control groups, including the following outcomes: Parents’ Depressive Symptoms; Parents’ Exercise Self-Efficacy; Parents’ Physical Activity; Children’s Physical Activity; Children’s Total Calories; Children’s Fiber Intake; Children Total Fat; Children’s Total Protein.

**Supplemental Table 1. Program Evaluation Form and Coding Sheet**

**Progress Evaluation Form (Intervenor to complete after each session)**

**Session #: _____ (Zoom__ or Phone call__) Family role: _____________ID: _____ Date:______**

| *Please assess and record parent’s stages of change and notes (if any)* | **Current stage**  (score 1-10) | **Special notes** |
| --- | --- | --- |
| Perceived **Importance** |  |  |
| Perceived **Readiness** |  |  |
| Perceived **Confidence** |  |  |
| Key Observations and Comments: | | |

*Please record* ***barriers/concerns*** *that were discussed in this session and by whom.*

| **Area of Evaluation:** | **Eating** | **Physical Activity** |
| --- | --- | --- |
| Lack of Money |  |  |
| Lack of Time |  |  |
| Lack of Motivation/Interest |  |  |
| Distance |  |  |
| Lack of Support |  |  |
| Pain/Discomfort |  |  |
| Lack of Knowledge |  |  |
| Other: |  |  |
| Key Observations and Comments: | | |

*Please record* ***motivators and strengths*** *that were discussed and by whom.*

| **Area of Evaluation:** | **Eating:** | **Physical Activity:** |
| --- | --- | --- |
| Family |  |  |
| Mental Health |  |  |
| Physical Health |  |  |
| Longer Lifespan |  |  |
| Better Opportunities |  |  |
| Other |  |  |
| Key Observations and Comments: | | |

***Short Term Goals*** *that were discussed and planned (X =Parent, O = Child)*

| **Area of Evaluation:** | **Eating:** | **Physical Activity** | **Specific notes** |
| --- | --- | --- | --- |
| Calories expenditure |  |  |  |
| Carbohydrates |  |  |  |
| Fats |  |  |  |
| Portion size |  |  |  |
| Exercise |  |  |  |
| Time Management |  |  |  |
| Weight |  |  |  |
| Others: |  |  |  |
| **Plan for the whole family.** |  | | |
| Key Observations and Comments: | | | |
| Education with Permission implemented: Yes ___ N0 ____ | | | |

**MM-based-MI Coding Sheet (by the Intervenor Peer after listening to the recording)**

**Audio #______________ session# __________Coder initial ________________ Date ____________**

| **MI Intervention Contents** | **Note** | **Rating (1-5 scale)** |
| --- | --- | --- |
| **Evocation** |  | 1 2 3 4 5  Low High |
| **Collaboration** |  | 1 2 3 4 5  Low High |
| **Autonomy/Support** |  | 1 2 3 4 5  Low High |
| **Direction** |  | 1 2 3 4 5  Low High |
| **Empathy** |  | 1 2 3 4 5  Low High |

**Intervenors’ Behavior Counts**

| **Health Coaching (information giving)** | | **Observational notes** |
| --- | --- | --- |
| **MI**  adherent | **Asking permission, affirm, emphasize control, support** |  |
| **MI**  Non-adherent | **Advise, confront, direct** |  |
| **Question**  (subclassify)  **Reflect**  (subclassify) | **Closed question** |  |
|  | **Open question** |  |
|  | **Simple** |  |
|  | **Complex** |  |
|  | **TOTAL REFLECTIONS:** |  |

**MM content rating**

| **MM contents** | **Note** | **Rating (1-5 scale)** |
| --- | --- | --- |
| **Acting with awareness** |  | 1 2 3 4 5  Low High |
| **Non-Judgmental** |  | 1 2 3 4 5  Low High |
| **Observe** |  | 1 2 3 4 5  Low High |
| **Non-reactivity** |  | 1 2 3 4 5  Low High |
| **Clearly Describe exercises** |  | 1 2 3 4 5  Low High |
| **Supportive** |  | 1 2 3 4 5  Low High |
| **Encourage regular practice** |  | 1 2 3 4 5  Low High |
| ***Additional observations related MM*** |  | |

**Supplemental Table 2: Intervention Program Evaluation**

| **Participants’ Questions (1‒5 scale)** | **N** | **Mean** | **SD** |
| --- | --- | --- | --- |
| 1. Are you satisfied with the way you were involved in discussions about you and your family members' lifestyles? | 20 | 4.40 | 4.40 |
| 1. Are you satisfied with the way you were assisted to maintain a positive outlook? | 20 | 4.45 | 4.45 |
| 1. Are you satisfied with the discussions you had about how to lead a mindful and healthier lifestyle? | 20 | 4.30 | 4.30 |
| 1. Are you satisfied with the way your questions were answered? | 20 | 4.60 | 4.60 |
| 1. Are you satisfied with the mindful exercises you were given on how to be mindful about family interactions? | 19 | 4.47 | 4.47 |
| 1. Were the mindful strategies/exercises helpful for you to manage your stress? | 20 | 4.25 | 4.25 |
| 1. As a result of taking part in the program, how much have your eating and physical activity improved? | 20 | 3.95 | 3.95 |
| 1. Was the time provided enough for you to learn the strategies? | 20 | 4.65 | 4.65 |
| 1. Would you recommend this to others? | 20 | 4.05 | 4.05 |
| 1. Was the presenter able to carry out the health education clearly? | 19 | 4.84 | 4.84 |
| 1. Was the educational material appropriate for your cultural background? | 20 | 4.75 | 4.75 |
|  | | | |
| **Open-ended questions** | | | |
| 1. What do you consider the strengths of this program? | | | |
| 1. In your opinion, what areas of the program could be improved? | | | |
| 1. What kind of suggestions do you have that could improve participants’ commitment to the program? | | | |
| 1. What kind of change would you like to make in order to be more relevant to your cultural background? | | | |

***Note*. 5-point scale:** 1= *not at all; 2 = a little, 3 = to some extent, 4* = *a good amount*, 5= *very much*

92.7% of intervention participants were satisfied with various aspects of intervention (rated ≥3 out of 5 points).
